# Association of Human Leukocyte Antigen (HLA) class II (*DRB1* and *DQB1*) alleles and haplotypes with Rheumatoid Arthritis in Sudanese patients

**DOI:** 10.3389/fimmu.2023.1178546

**Published:** 2023-06-22

**Authors:** Adil Ahmed Ali, Khalid Eltahir Khalid, Somaya Elhaj Mohammed, Mohammed Salman Akhtar, Osman Khalafalla Saeed

**Affiliations:** ^1^ Department of Biochemistry and Nutrition, Faculty of Medicine, University of Gezira, Wad Medani, Sudan; ^2^ Department of Basic Medical Sciences, Faculty of Applied Medical Sciences, Al-Baha University, Al-Baha, Saudi Arabia; ^3^ Department of Pathology, Faculty of Medicine, University of Gezira, Wad Medani, Sudan; ^4^ Department of Internal Medicine, Faculty of Medicine, University of Gezira, Wad Medani, Sudan

**Keywords:** rheumatoid arthritis, HLA-DRB1, HLA-DQB1, anti-CCP, rheumatoid factor

## Abstract

The aim of this study was to determine the Human Leukocyte Antigen (HLA) class II (*DRB1* and *DQB1*) alleles and haplotype frequency in Rheumatoid Arthritis **(**RA) in the Sudanese population. The frequency of *HLA-DRB1* and -*DQB1* alleles and *DRB1-DQB1* haplotypes were determined in 122 RA patients and 100 controls. HLA alleles were genotyped by the polymerase chain reaction-sequence specific primers (PCR-SSP) method. In RA patients, HLA-DRB1*04 and *10 alleles were high in frequency (9.6% vs 14.2%, *P* = 0.038 and *P* = 0.042, respectively), and dependently on anti-citrullinated protein antibodies (ACPAs) seropositivity (*P* = 0.044 and *P* = 0.027, respectively). In contrast, the frequency of the HLA-DRB1*07 allele was significantly low in patients than in controls (11.7% vs 5.0%, *P* = 0.010). Moreover, the HLA-DQB1*03 allele was strongly associated with RA risk (42.2%, *P* = 2.2x10^-8^), whereas, HLA-DQB1*02 and *06 showed protective effects against RA (23.1% and 42.2%, *P* = 0.024 and *P* = 2.2x10^-6^, respectively). Five different HLA haplotypes, DRB1*03-DQB1*03 (*P* = 0.00003), DRB1*04-DQB1*03 (*P* = 0.00014), DRB1*08-DQB1*03 (*P* = 0.027), DRB1*13-DQB1*02 (*P* = 0.004), and DRB1*13-DQB1*03 (*P* = 3.79x10^-8^) were significantly associated with RA risk, while 3 protective haplotypes, DRB1*03-DQB1*02 (*P_c_* = 0.008), DRB1*07-DQB1*02 (*P_c_* = 0.004), and DRB1*13-DQB1*06 (*P_c_* = 0.02) were identified. This is the first study determining the association between HLA class II alleles and haplotypes and RA risk in our population.

## 1 Introduction

Rheumatoid arthritis (RA) is a systemic chronic inflammatory disease of unknown etiology, characterized by multifactorial etiology and complex genetic background, particularly with Human Leukocyte Antigen (HLA) (1). The exact etiology of RA is unknown, although the pathogenesis is critically influenced by genetic and environmental factors. The genetic components, particularly for HLA-DR/DQ genotypes were extensively studied worldwide and proved to contribute to RA risk (2). Their rate of contribution to RA is varied in different studies and between different populations. In general, RA patients who carried certain HLA-DR/DQ alleles are subjected to a high risk of developing the disease in different populations (3–5), including sub-Saharan African populations (6, 7). There is extensive evidence that some HLA-DRB1 alleles, including HLA-DRB1*01, HLA-DRB1*04, HLA-DRB1*09, HLA-DRB1*10, and HLA-DRB1*14 implicated with RA susceptibility in different populations (7–11).

The serological anti-citrullinated protein antibodies (ACPA) and the rheumatoid factor (RF) have been extensively studied and reported as important diagnostic and prognostic biomarkers for RA and were also found to be associated with more aggressive and erosive disease. ACPA was found more specific and sensitive for RA diagnosis and a better predictor of poor prognostic features such as progressive joint destruction and has been strongly associated with HLA class 11 molecules, like HLA-DRB1 (8, 12).

Sudan is a large country of high effective population size and traditional societies that have diverse linguistic, ethnic, social, cultural, and religious characteristics. The manifestation of the impact of culture in disease burden with regard to consanguinity has been associated with genetic disorders, particularly those of autosomal recessive nature such as sickle cell mutation (HbS) among the ‘Baggara’, and with lactase persistence (LP) mutations among the ‘Beja’ and ‘Fulani’ of western Sudan (9, 10). In our population, with regard to different ethnicities and consanguineous marriages, no study relating RA and genetic associations has been yet conducted and no data on RA prevalence, especially with RF or anti-CCP positivity, are available. Therefore, this study was conducted for the first time to investigate the frequency of *HLA-DRB/DQ* alleles and haplotypes and their association with RA, as this might emphasize the nature, susceptibility, and prognosis of the disease in our population.

## 2 Materials and methods

### 2.1 Study subjects

This is a cross-sectional study, comprising 122 RA patients (mean age, 44.95±14.03 yrs; 106 female, 16 male) diagnosed in the rheumatology clinics at Ibrahim Malik & The Academy Teaching Hospitals, in Khartoum state-Sudan. Sudan’s Khartoum state, in which the study was conducted, is the capital, fulminated by different ethnic groups that equally share easy access to health facilities that are provided via primary and tertiary health care. The quality of life in the state is quite high as to other regions, where the highest abundance of health facilities exists.

All patients were clinically examined by two rheumatologists and were assessed based on the American College of Rheumatology Criteria (ACR) Board of Directors and the European League Against Rheumatism (EULAR) Executive Committee (13). The demographic, clinical information, and laboratory profiles were indicated in the clinical interview form as shown in **Table 1**. The control group included 100 non-related healthy volunteers (mean age, 43.06±10.51 years; 89 female, 11 male), with no apparent disease, no family history of RA or other autoimmune diseases, and were from the same hospitals, departments, and geographical areas. All participants were notified of the study purposes and objectives, and written consent was obtained before the inclusion applied, directly from adults or guardians of children under 18 years of age. The study protocol was approved by the University of Gezira Ethical Committee and performed according to the ethical guidelines of the Helsinki Declaration.

**Table 1:** Baseline characteristics in RA patients and healthy controls.

Parameter		RA Patients (n = 122)		Healthy Controls (n = 100)	
		Female N (%)	Male N (%)	Female N (%)	Male N (%)
**Number (%)**		106 (86.9)	16 (13.1)	89 (89.0)	11 (11.0)
**Age, mean±SD**		44.97±14.00	44.81±14.64	43.62±9.89	38.55±14.37
**Age Groups/years**	10-19	4 (3.8)	1 (6.3)	3 (3.4)	0
	20-29	10 (9.4)	1 (6.3)	12 (13.5)	0
	30-39	18 (17.0)	1 (6.3)	27 (30.3)	6 (54.5)
	40-49	31 (29.2)	6 (37.5)	30 (33.7)	3 (27.3)
	50-59	23 (21.7)	4 (25.0)	14 (15.7)	2 (18.2)
	60-69	14 (13.2)	3 (8.8.)	0	0
	70-79	5 (4.7.)	0	3 (3.4)	0
	80-89	1 (0.9)	0	0	0
**Disease Duration ± SD years**		3.62±4.37	3.15±4.40	NA	NA
**Family History of RA**	Yes	47 (44.3)	12 (75.0)	0	0
	No	59 (55.7)	4 (25.0)	89 (100.)	11 (100.0)
**Degree of Relatives**	No	59 (55.7)	4 (25.0)	0	0
	First	35 (33.0)	8 (50.0)	0	0
	Second	12 (1.3)	4 (25.0)	0	0
**Residence**	Rural	17 (16.0)	9 (56.3)	3 (.4)	0
	Urban	89 (84.0)	7 (43.2)	86 (96.4)	11 (100.0)
**Smoking**	Yes	2 (1.9)	7 (43.8)	0	5 (45.5)
	No	104 (98.1)	9 (56.2)	89 (100.0)	6 (54.5)

NA, not applicable; RA, rheumatoid arthritis; N, number; %, percentage; SD, standard deviation.

### 2.2 HLA-DR genotyping

Genomic DNA was extracted from peripheral venous blood using QIA amp^®^ DNA mini kit (Qiagen CA, USA) according to the manufacturer’s instructions.

HLA class II *DRB1-DQB1* genotyping was carried out by polymerase chain reaction (PCR) with sequence-specific primers (SSP) using low-resolution kits (Rose, HLA-SSP Typing Kits, Germany). The PCR amplification of DNA was performed using Applied Biosystem 9700 thermo-cycler (Thermo Fisher, UK), and the procedure was carried out according to the manufacturer’s instructions. The obtained DNA fragments were photographed, edited, and documented on 2% agarose gel with 0.5μg/ml ethidium bromide added, using a gel documentation system, and the typing genotypes were obtained using HLA software provided by Rose Company (R.O.S.E. Europe GmbH).

### 2.3 Immunological measurements

Serum ACPA was measured using anti-CCP IgG antibodies, detected by Immunoscan CCPlus^®^ ELISA kits (Euro Diagnostic, AB-Malmo, Sweden), as per the manufacturer’s instructions. ACPA concentration >25 U/ml was considered positive. Serum IgM rheumatoid factor (RF) and c-reactive protein (CRP) were assessed using an automated chemistry analyzer (Cobas Mira Plus, Roche, Basel, Switzerland) and rapid latex agglutination test (NS Bio-Tec, Egypt), respectively. CRP level <10 mg/L (0-9.9 mg/L) was considered normal, while the presence of RF agglutination indicates a positive test.

### 2.4 Statistical analysis

The statistical analysis was conducted by using SPSS software, version 23 (SPSS Inc., Chicago, IL, USA). Categorical variables were presented as counts and percentages. The independent sample ***t*-**test was used to compare the mean differences of quantitative variables between patients and controls. The significant differences in allele frequencies (AF) of *DRB1* and *DQB1* were compared between patients and controls by using a chi-square test or Fisher’s exact test for discrete data. Odds ratios (ORs) and 95% confidence intervals (CIs) were calculated as indicated. Two-locus associations and haplotype frequencies (HF) were determined by direct counting of samples with a particular combination of alleles, and the significance of these associations was determined by a chi-square test with Yates’ correction. Bonferroni correction was used for alleles and haplotypes with frequencies >5% and applied when the *P*-value is significant. Corrected probability values (*Pc*) were calculated by multiplying individual *P*-values by the number of comparisons made at the allele or haplotype levels: 8 for *HLA-DRB1* alleles and 3 for *DRB1-DQB1* haplotypes. In our data analysis, a *P*-value less than 0.05 was considered significant.

## 3 Results

### 3.1 Demographic and clinical characteristics of RA patients

The demographic data and the clinical features of 122 patients diagnosed with RA are presented in **Table 1**. Mainly females are predominant in the study subjects. A high frequency of RA patients was found in the age group between 40-59 years. Almost half of the patients (48.4%) were having a family history of RA, out of them 72.9% as first-degree relatives. The RA clinical picture indicates that the majority (68.0%) suffered from morning stiffness, and half of the patients were positive for ACPA (52.5%) and CRP (45.9%). Joint pain was mostly in the wrist, fingers, knee & hands. Ulnar deviation is the most frequent deformity (26.2%), followed by Boutonniere deformity (14.8%) (**Table 2**).

**Table 2:** RA Clinical characteristics and deformities.

Clinical Characteristics and Deformities	RA Patients (n=122)	
	Yes N (%)	NO N (%)
Clinical characteristics & Lab results		
**Morning Stiffness**	83 (68.0)	39 (32.0)
**ACPA+**	64 (52.5)	58 (47.5)
**RF+**	30 (24.6)	92 (75.4)
**CRP+**	56 (45.9)	66 (54.1)
**ACPA+/RF+**	24 (19.7)	98 (80.3)
Joints affected by RA		
**Fingers**	104 (85.2)	18 (14.8)
**Hands**	103 (84.4)	19 (15.6)
**Wrists**	110 (90.2)	12 (9.8)
**Elbows**	78 (63.9)	44 (36.1)
**Shoulders**	74 (60.7)	48 (39.3)
**Neck**	40 (32.8)	82 (67.2)
**Back**	33 (27.0)	89 (73.0)
**Toes**	64 (52.5)	58 (47.5)
**Feet**	79 (64.8)	43 (35.2)
**Ankles**	88 (72.1)	34 (27.9)
**Knees**	104 (85.2)	18 (14.8)
**Hips**	33 (18.9)	99 (81.1)
Deformities		
**Boutonniere Deformity**	18 (14.8)	104 (85.2)
**Ulnar deviation**	32 (26.2)	90 (73.8)
**Swan-Neck Deformity**	17 (13.9)	105 (86.1)
**Z thumb**	17 (13.9)	105 (86.1)
**Presence of Rheumatic Nodules**	8 (6.6)	114 (93.4)

RA, rheumatoid arthritis; N, number; %, percentage; ACPA, anti-citrullinated protein antibodies; RF, rheumatoid factor; CRP, C-reactive protein.

### 3.2 *DRB1* and *DQB1* alleles analysis

The distribution of *HLA-DRB1* and *HLA-DQB1* alleles in our study subjects is shown in **Table 3**. In patients, the most frequent *HLA-DRB1* alleles were *13 (20.4%), *10 (14.2%), and *15 (12.5%), while HLA-DRB1*13 (23.5%), *11 (22.2%), and *07 (11.7%) were in controls. HLA-DRB1*04 and *10 frequency were significantly higher in patients than in controls [9.6% vs 5.1%, *P* = 0.038, OR (95% CI) = 0.47 (0.32-0.54) and 14.2% vs 8.2%, *P* = 0.042, OR (95% CI) = 1.86 (0.99-3.48)], and was associated with ACPA positivity [22.2% vs 8.8%, *P* = 0.044, OR (95% CI) = 2.97 (1.00-8.87) and 23.8% vs 8.8%, *P* = 0.027, OR (95% CI) = 3.25 (1.10-9.62), respectively] (**Table 4**). In contrast, the HLA-DRB1*07 allele was significantly lower (*P* = 0.010) in patients (5.0%) than in controls (11.7%).

**Table 3:** HLA-DRB1 and DQB1 allele frequencies in RA patients and healthy controls^a^.

Allele HLA-DRB1	Healthy Controls n = 196		RA Patients n = 240		Patients vs Controls	
	n	AF	n	AF	*P*-value	P_c_
*01	13	6.6	13	5.4	0.591	
*03	21	10.7	23	9.6	0.697	
*04	10	5.1	23	9.6	**0.038^b^**	0.304
*07	23	11.7	12	5.0	**0.010^c^**	0.080
*08	19	9.7	27	11.3	0.599	
*09	02	1.0	01	0.4	0.424	
*10	16	8.2	34	14.2	**0.042^d^**	0.336
*11	24	22.2	26	10.8	0.645	
*13	46	23.5	49	20.4	0.442	
*14	0	0	01	0.4	NA	
*15	21	10.7	30	12.5	0.564	
*16	01	0.5	01	0.4	0.698	
HLA-DQB1						
*02	46	23.1	36	14.8	**0.024^e^**	0.072
*03	35	17.6	103	42.2	**2.2x10^-8f^**	**6.6x10^-8^**
*04	0	0	02	0.8	0.303	
*05	32	16.1	46	18.9	0.446	
*06	84	42.2	57	23.4	**2.2x10^-6g^**	**6.6x10^-6^**
*07	1	0.5	0	0	0.449	
*13	1	0.5	0	0	0.449	

NA, not applicable; AF, allele frequency; CI, confidence interval; OR, odds ratio; n, number of individuals, RA, rheumatoid arthritis; P_c_ (P corrected), P -value after Bonferroni correction with the number of comparison = 9 for DRB1 alleles or 4 for DQB1 alleles. Significant associations are indicated in bold. **^a^** Only DRB1/DQB1 alleles with frequencies >5% and significant values in either cases or controls were analyzed for Bonferroni correction; Degree of freedom = 1. **^b^** OR (95% CI) = 0.47 (0.32-0.54); **^c^** OR (95% CI) = 0.40 (0.19-0.82); **^d^** OR (95% CI) = 1.86 (0.99-3.48); **^e^** OR (95% CI) = 0.58 (0.34-9.30); **^f^** OR (95% CI) = 3.43 (2.19-5.34); **^g^** OR (95% CI) = 0.42 (0.28-0.63).

**Table 4:** HLA-DRB1 and DQB1 allele frequencies in ACPA/RF^+^ and ACPA/RF^-^ RA patients^a^.

Allele HLA-DRB1	RA Patients Subgroups								ACPA+ vs ACPA-		RF+ vs RF-	
	ACPA+ n = 63		ACPA- n = 57		RF+ n = 29		RF- n = 91					
	n	AF	n	AF	n	AF	n	AF	*P*-value	P_c_	*P*-value	P_c_
*01	06	9.5	05	8.8	04	13.8	07	7.7	0.887		0.458	
*03	09	14.3	07	12.3	03	10.3	13	14.3	0.747		0.758	
*04	14	22.2	05	8.8	04	13.8	15	16.5	**0.044^b^**	0.352	1.000	
*07	04	6.3	05	8.8	03	10.3	06	6.6	0.615		0.450	
*08	03	4.8	14	24.6	02	6.9	15	16.5	**0.002^c^**	**0.016**	0.239	
*09	01	1.6	0	0	0	0	01	1.1	1.00		1.000	
*10	15	23.8	05	8.8	07	24.1	13	14.3	**0.027^d^**	0.216	0.254	
*11	02	3.2	07	12.3	02	6.9	07	7.7	0.059		1.000	
*13	04	6.3	05	8.8	03	10.3	06	6.6	0.615		0.450	
*14	0	0	0	0	0	0	0	0	NA		NA	
*15	05	7.9	04	7.0	01	3.4	08	8.8	0.849		0.686	
*16	0	0	0	0	0	0	0	0	NA		NA	
HLA-DQB1												
*02	15	23.4	14	24.1	09	30.0	20	24.1	0.928		0.356	
*03	31	50.0	27	48.3	15	50.0	45	48.3	0.849		0.918	
*04	0	0	0	0	0	0	0	0	NA		NA	
*05	13	20.3	11	19.0	06	20.0	18	19.0	0.852		0.959	
*06	4	6.3	5	8.6	0	0	09	8.6	0.735		0.111	
*07	0	0	0	0	0	0	0	0	NA		NA	
*13	0	0	0	0	0	0	0	0	NA		NA	

NA, not applicable; AF, allele frequency; CI, confidence interval; OR, odds ratio; n, number of individuals, RA, rheumatoid arthritis; ACPA, anti-citrullinated protein antibodies; RF, rheumatoid factor; P_c_ (P corrected), P -value after Bonferroni correction with the number of comparisons = 9 for DRB1 alleles or 4 for DQB1 alleles. Significant associations are indicated in bold. **^a^** Only DRB1/DQB1 alleles with frequencies >5% and significant values in either cases subgroups were analyzed for Bonferroni correction; Degree of freedom = 1. **^b^** OR (95% CI) = 2.97 (1.00-8.87); **^c^** OR (95% CI) = 0.39 (0.19-0.80); **^d^** OR (95% CI) =3.25 (1.10-9.62).

Among ACPA seronegative patients, HLA-DRB1*08 frequency was significantly higher than in ACPA seropositive counterpart [24.6% vs 4.8%, *P* = 0.002, OR (95% CI) = 0.40 (0.19-0.82)], which remained significant after Bonferroni correction (*P*_c_ = 0.016).

No associations were found between RF antibody and *HLA-DRB1* and *HLA-DQB1* alleles and haplotypes (**Table 4**).

The most frequent *HLA-DQB1* alleles in patients were HLA-DQB1*03 (42.2%) and *06 (23.4%), while in controls, were HLA-DQB1*06 (42.2%) and *02 (23.1%). HLA-DQB1*03 allele was significantly higher in frequency and associated with RA risk [42.2% vs 17.6%, *P* = 2.2x10^-8^; OR (95% CI) = 3.43 (2.19-5.34)], this association persisted after multiple comparisons (*P*_c_ = 6.6x10^-8^). In contrast, HLA-DQB1*02 and *06 were significantly higher in frequency in controls and may confer protection [23.1% vs 14.8%, *P* = 0.024, OR (95% CI) = 0.58 (0.34-9.30); 42.2% vs 23.4%, *P* = 2.2x10^-6^, OR (95% CI) = 0.42 (0.28-0.63), respectively] (**Table 3**). HLA-DQB1*06 significance remained following Bonferroni correction (*P*_c_ = 6.6x10^-6^).

### 3.3 DRB1-DQB1 haplotypes analysis

As shown in **Table 5**, 13 *HLA-DRB1* and *HLA-DQB1* haplotypes were identified in our study subjects. In patients as compared with controls, HLA-DRB1*03-DQB1*03 [2.8% vs -%, *P* = 0.00003, OR (95% CI) = 2.54 (2.20-2.93)], HLA-DRB1*04-DQB1*03 [3.2% vs 0.5%, *P* = 0.00014, OR (95% CI) = 10.85 (2.42-48.65)], HLA-DRB1*13-DQB1*02 [1.4% vs -%, *P* = 0.004, OR (95% CI) = 2.46 (2.14-2.83)], and HLA-DRB1*13-DQB1*03 [5.6% vs 0.2%, *P* = 3.79x10^-8^, OR (95% CI) = 41.11 (5.48-308.46)] were associated with RA risk, each of which remained significant after correction of multiple comparisons (*P*_c_ = 0.0003, *P*_c_ = 0.0001, *P*_c_ = 0.032, and *P*_c_ = 3.3x10^-9^, respectively). On the other hand, HLA-DRB1*03-DQB1*02 [0.7% vs 4.6%, *P* = 0.004, OR (95% CI) = 0.19 (0.06-0.65), HLA-DRB1*07-DQB1*02 [1.3% vs 5.1%, *P* = 0.002, OR (95% CI) = 0.17 (0.05-0.58)], and HLA-DRB1*13-DQB1*06 [3.9% vs 10.4%, *P* = 0.010, OR (95% CI) = 0.45 (0.24-0.83)] haplotypes were significantly low in patients than in controls and remained significant when Bonferroni correction applied (*P*_c_ = 0.008, *P*_c_ = 0.004 and *P*_c_ = 0.02 respectively) (**Table 5**).

**Table 5:** HLA-DRB1/DQB1 haplotype frequencies in RA patients and healthy controls^a^.

DRB1/DQB1 Haplotype	Health Controls n =196		RA Patients n = 240		Patients vs Controls	
	n	HF	n	HF	*P*-value	P_c_
*01/05	13	3.0	03	0.7	0.055	0.11
*03/02	20	4.6	03	0.7	**0.004^b^**	**0.008**
*03/03	0	0	12	2.8	**0.00003^c^**	**0.00006**
*04/03	02	0.5	14	3.2	**0.00014^d^**	**0.0003**
*04/06	05	1.2	02	0.2	0.703	
*07/02	22	5.1	03	1.3	**0.002^e^**	**0.004**
*08/03	06	1.4	12	2.8	**0.027^f^**	0.054
*10/05	12	2.8	09	2.1	0.919	
*11/03	24	5.6	09	2.1	0.073	
*13/02	0	0	06	1.4	**0.004^g^**	**0.008**
*13/03	01	0.2	24	5.6	**3.79x10^-8h^**	**7.58x10^-8^**
*13/06	45	10.4	17	3.9	**0.010^i^**	**0.02**
*15/06	21	4.9	09	2.1	0.167	

HF, haplotype frequency; CI, confidence interval; OR, odds ratio; n, number of individuals, RA, rheumatoid arthritis; P_c_ (P corrected), P -value after Bonferroni correction with the number of comparison = 3 for DRB1/DQB1 haplotype. Significant associations are indicated in bold.

**^a^** Only DRB1/DQB1 haplotypes with frequencies >5% in either cases or controls were analyzed for Bonferroni correction, Degree of freedom = 1. **^b^** OR (95% CI) = 0.19 (0.06-0.65); **^c^** OR (95% CI) = 2.54 (2.20-2.93); **^d^** OR (95% CI) = 10.85 (2.42-48.65); **^e^** OR (95% CI) = 0.17 (0.05-0.58); **^f^** OR (95% CI) = 2.97 (1.09-8.15); **^g^** OR (95% CI) = 2.46 (2.14-2.83); **^h^** OR (95% CI) = 41.11 (5.48-308.46); **^i^** OR (95% CI) = 0.45 (0.24-0.83).

Furthermore, HLA-DRB1*08-DQB1*03 frequency was significantly high in patients [2.8% vs 1.4%, *P* = 0.027, (95% CI) = 2.97 (1.09-8.15)] and was found to be associated with ACPA seronegativity [OR (95% CI) = 0.09 (0.01-0.70), *P* = 0.006, *P*_c_ = 0.012] (**Table 6**). No association was found between *HLA-DRB1/DQB1* haplotypes and RF antibodies (**Table 6**).

**Table 6:** HLA-DRB1/DQB1 haplotype frequencies in ACPA/RF^+^ and ACPA/RF^-^ RA patients^a^.

DRB1/DQB1 Haplotype	RA Patients Subgroups								ACPA+ vs ACPA-		RF+ vs RF-	
	ACPA+ n = 63		ACPA- n = 57		RF+ n = 29		RF- n = 91					
	n	HF	n	HF	n	HF	n	HF	*P*-value	P_c_	*P*-value	P_c_
*01/05	0	3.5	02	3.5	0	0	02	2.2	0.224		1.000	
*03/02	01	1.6	01	1.8	01	3.4	01	1.1	1.000		0.426	
*03/03	05	7.9	05	8.8	02	6.9	08	8.8	1.000		1.000	
*04/03	09	14.3	03	5.3	03	10.3	09	9.9	0.100		1.000	
*04/06	0	0	01	1.8	0	0	01	1.1	0.471		1.000	
*07/02	01	1.6	02	3.5	01	3.4	02	2.2	0.604		0.567	
*08/03	01	1.6	09	15.8	01	3.4	09	9.9	**0.006^b^**	**0.012**	0.448	
*10/05	04	6.3	02	3.5	03	10.3	03	3.3	0.682		0.151	
*11/03	02	3.2	02	3.5	01	3.4	03	3.3	1.000		1.000	
*13/02	0	0	03	5.3	01	3.4	02	2.2	0.065		0.567	
*13/03	03	4.8	02	3.5	01	3.4	04	4.4	1.000		1.000	
*13/06	0	0	0	0	0	0	0	0	NA		NA	
*15/06	0	0	01	1.8	0	0	01	1.1	0.471		1.000	

NA, not applicable; HF, haplotype frequency; CI, confidence interval; OR, odds ratio; n, number of individuals, RA, rheumatoid arthritis; ACPA, anti-citrullinated protein antibodies; RF, rheumatoid factor; P_c_ (P corrected), P -value after Bonferroni correction with the number of comparison = 3 for DRB1/DQB1 haplotype. Significant associations are indicated in bold.

**^a^** Only DRB1/DQB1 haplotypes with frequencies >5% in either cases subgroups were analyzed for Bonferroni correction, Degree of freedom = 1. **^b^** OR (95% CI) = 0.09 (0.01-0.70).

## 4 Discussions

The most important genetic factors associated with RA are the Human Leukocyte Antigen (HLA) linked genes, accounting for approximately 30% of the total genetic contribution for RA susceptibility (14). The *HLA-DRB1* locus is highly polymorphic and confers more risk for RA than any other locus (15). Susceptibility for developing rheumatoid arthritis (RA) is associated with particular *HLA-DRB1* alleles like HLA- DRB1*04, HLA-DRB1*01, and HLA-DRB1*10 (16).

In our study cases, HLA-DRB1*04 and *10 were the most frequent alleles, which is consistent with studies in populations with different ethnicity (11, 17–23). Two small genetic studies have been performed in West/Middle Africa, showing the risk of developing RA was associated with HLA-DRB1*10 but not *04 (6, 24). Compared to people of European descent, African Americans are thought to have a lower prevalence of RA, lower frequency of the highest risk *HLA-DRB1* classical alleles (e.g. *04:01, *04:04), and lower effect size of high-risk alleles with RA (15).

We also found that both alleles were statistically associated with ACPA seropositivity, which is consistent with studies in populations with different ethnicity (7, 25–28). Furthermore, strong evidence suggests that some *HLA-DRB1* alleles, including HLA-DRB1*01, *04, and *10, were associated with the structural severity of RA, and were likely related to the production of ACPA by influencing the antigen presentation (29, 30).

In contrast, the HLA-DRB1*07 allele was found significantly increased in controls as compared with RA patients. This finding was consistent with studies from Algeria (27), Tunis (31, 32), Turkey (33), Finland (34), and Slovakia (35), suggesting that DRB1*07 confers a protective effect against RA development. On the contrary, the HLA-DRB1*07 allele was found common in both cases and controls in Bangladeshi (17) and Pakistani (36) populations.

On the other hand, our data showed that the significant frequent alleles of *HLA-DQB1* identified in RA patients were HLA-DQB1*03 (P = 0.000, OR = 3.43), whereas in controls, was HLA-DRQB1*02 (P = 0.02, OR = 0.58) and *06 (P = 0.00, OR = 0.42).

HLA-DRQ1*02 and *06 were found protective against RA in the Albanian population (28), while in the Kurdi population, these alleles showed a susceptible effect to RA (37). Other studies in the Turkish population failed to prove any association (33).

In this study, HLA-DRB1*03/DRB1*03, HLA-DRB1*04/DRB1*03, HLA-DRB1*08/DRB1*3, HLA-DRB1*13/DRB1*02, and HLA-DRB1*13/DRB1*03 haplotypes, were associated with RA risk. A previous study on females with RA found that HLA-DRB1*03/DRB1*03 was associated with the development of RA, while HLA-DRB1*13/DRB1*03 represents a protective effect (38), other studies found that the haplotype HLA-DQB1*3 was linked to HLA-DRB1*09:01 or *04 and the haplotype HLA-DQB*05 linked to HLA-DRB1*01:01, *01:02, *01:03, and *10:01, and they were positively associated with RA in Caucasians (34). Collectively, the controversial results in HLA genotyping are strongly associated with the genetic makeup, owing to the differences in ethnicity between populations.

In this study, the level of biochemical markers such as ACPA, CRP, and ESR was found significantly high in RA patients as compared with the controls, which was consistent with other studies (39–42), and may indicate that the maintenance of process activity in RA patients as a result of acute phase reactants, the acceleration of sedimentation, and high CRP (41). Nevertheless, compared to anti-CCP, our data showed no significant association was found between HLA genotypes and RF. This finding is similar to the studies conducted in Turkish and Saudi populations, where the association was found with positive anti-CCP and not with RF (43, 44). This is not surprising as anti-CCP is more specific than RF, and seemed to play a pivotal role in the pathogenesis of RA.

Our data showed that HLA-DRB1*03/DRB1*02, HLA-DRB1*07/DRB1*02, and HLA-DRB1*13/DRB1*06 haplotypes confer a protective effect against RA. The protective effect of the HLA-DRB1*07/DRB1*02 haplotype was stated earlier in a population from Pakistan, who reported a negative association with RA (36). In parallel, a Moroccan study reported that the frequency of DRB1*07-DQB1*02 and DRB1*13-DQB1*06 haplotypes was decreased in RF-positive patients compared to controls (45). The HLA-DRB1*03/DRB1*02 protective effect has been observed in multiple autoimmune disorders, such as celiac disease (46) and type 1 diabetes (47), but not in RA.

## 5 Conclusions

The present study highlighted and reported for the first time the association of HLA class II (*DRB1* and *DQB1*) alleles and haplotypes and the risk of developing RA in our population. HLA-DRB1*04, *10, and HLA-DQB1*03 were found associated with RA risk, while HLA-DRB1*07, HLA-DQB1*02, and *06 showed protection against RA. On the other hand, HLA-DRB1*03-DQB1*03, HLA-DRB1*04-DQB1*03, HLA-DRB1*08-DQB1*03, and HLA-DRB1*13-DQB1*02 haplotypes were associated with RA risk, whereas HLA-DRB1*03-DQB1*02, DRB1*07-DQB1*02, and HLA-DRB1*13- DQB1*06 confers protection.

## Limitation

We could not run the high-resolution genotyping due to cost constraints. Furthermore, the study sample size makes the degree of significance to determine the genetic risk relatively weak. More hands-on studies with large sample sizes and the use of high-resolution genotyping are needed to verify our findings.

## Data availability statement

The original contributions presented in the study are included in the article/supplementary materials. Further inquiries can be directed to the corresponding authors.

## Ethics statement

The studies involving human participants were reviewed and approved by University of Gezira Ethical Committee. The patients/participants provided their written informed consent to participate in this study.

## Author contributions

All authors contributed to the study perception and design; AA collected and analyzed the data with input from KK; AA, KK, and SM wrote the initial draft of the manuscript; KK, SM, MA, and OS revised and approved the final draft. All authors contributed to the article and approved the submitted version.
